# Epidermal Growth Factor (EGF) Augments the Invasive Potential of Human Glioblastoma Multiforme Cells via the Activation of Collaborative EGFR/ROS-Dependent Signaling

**DOI:** 10.3390/ijms21103605

**Published:** 2020-05-20

**Authors:** Maciej Pudełek, Kamila Król, Jessica Catapano, Tomasz Wróbel, Jarosław Czyż, Damian Ryszawy

**Affiliations:** Department of Cell Biology, Faculty of Biochemistry, Biophysics and Biotechnology, Jagiellonian University, Gronostajowa 7, 30-387 Kraków, Poland; maciej.pudelek@student.uj.edu.pl (M.P.); Kamilajulia.krol@student.uj.edu.pl (K.K.); 1catapanojessica@gmail.com (J.C.); 1tomaszwrobel@gmail.com (T.W.); jarek.czyz@uj.edu.pl (J.C.)

**Keywords:** glioblastoma multiforme, epidermal growth factor, invasion, EGFR, ROS

## Abstract

Abnormal secretion of epidermal growth factor (EGF) by non-neuronal cells (e.g., glioma-associated microglia) establishes a feedback loop between glioblastoma multiforme (GBM) invasion and a functional disruption of brain tissue. Considering the postulated significance of this vicious circle for GBM progression, we scrutinized mechanisms of EGF-dependent pro-invasive signaling in terms of its interrelations with energy metabolism and reactive oxygen species (ROS) production. The effects of EGF on the invasiveness of human glioblastoma T98G cells were estimated using time-lapse video microscopy, immunocytochemistry, cell cycle assay, immunoblot analyses, and Transwell^®^ assay. These techniques were followed by quantification of the effect of EGFR (Epidermal Growth Factor Receptor) and ROS inhibitors on the EGF-induced T98G invasiveness and intracellular ROS, ATP, and lactate levels and mitochondrial metabolism. The EGF remarkably augmented the proliferation and motility of the T98G cells. Responses of these cells were accompanied by cellular rear–front polarization, translocation of vinculin to the leading lamellae, and increased promptness of penetration of micropore barriers. Erlotinib (the EGFR inhibitor) significantly attenuated the EGF-induced T98G invasiveness and metabolic reprogramming of the T98G cells, otherwise illustrated by the increased mitochondrial activity, glycolysis, and ROS production in the EGF-treated cells. In turn, ROS inhibition by N-acetyl-L-cysteine (NAC) had no effect on T98G morphology, but considerably attenuated EGF-induced cell motility. Our data confirmed the EGFR/ROS-dependent pro-neoplastic and pro-invasive activity of EGF in human GBM. These EGF effects may depend on metabolic reprogramming of GBM cells and are executed by alternative ROS-dependent/-independent pathways. The EGF may thus preserve bioenergetic homeostasis of GBM cells in hypoxic regions of brain tissue.

## 1. Introduction

Despite the development of new oncological regimens, glioblastoma multiforme (GBM) treatment represents an indisputable challenge for contemporary neuro-oncology [1]. The clinical picture of GBM is often characterized by rapid development of invasiveness accompanied by swift recurrences after therapeutic cycles [2,3,4,5]. Furthermore, the anatomy of brain tissue causes difficulties with drug administration [6,7]. Therefore, it is not surprising that the median survival of diagnosed GBM patients ranges between 12 and 14 months, and has not changed considerably over the years [1,8,9]. Recently, numerous growth factors (EGF, TGF-β) and cytokines (e.g., IL-6) have been reported to support tumor growth and spreading [10,11]. In particular, the epidermal growth factor (EGF) is secreted by glioma-associated microglia and macrophages (GAMs), which infiltrate the GBM-violated brain tissue continuum and participate in paracrine loops within glioblastoma niches [10,12]. Accordingly, EGFR signaling seems to be crucial for GBM invasion and progression [13]. Almost 50% of diagnosed gliomas show amplification of the constitutively active EGF receptor (EGFR type III or EGFRvIII [13]). However, expression of functional ligand-binding EGF receptors in GBM cells also seems to play a pivotal role in GBM-related pathogenesis, but the details of the EGF-related signaling involved in GBM cell invasion still remain elusive [13,14,15,16].

The disruption of the neural tissue continuum can result from the GBM progression or may be caused by a surgical intervention (post-traumatic GBM [17,18,19]). EGF secretion plays an essential role in brain tissue healing and regeneration mechanisms [20,21,22], determining microglial migration to GBM lesions 10,20]). Numerous processes can determine invasion-related GBM cell reactions to the EGF [23,24]. In particular, glial-to-mesenchymal transition (GMT), which is an analogue of the epithelial-to-mesenchymal transition (EMT) in non-glial tumors [25,26,27,28,29], contributes to the microevolution of GBM malignancy. It is controlled by a set of transcription factors (Snail-1, Twist, ZEB-1, and Slug [28]) and leads to activation of GBM cell migration via effects on cell mechanics, bioenergetic homeostasis, and the secretome. Thus, it enhances the invasive potential of GBM cells and their ability to invade healthy tissues [28,29]. These effects are regulated by the “secondary executors”, which includes a cytoplasmic pool of connexins [30,31], and by precisely controlled gradients of reactive oxygen species [32,33,34], which play a pivotal role as migration activity modulators. Notably, EGFR signaling is also involved in bioenergetic homeostasis, contributing to metabolic reprogramming of GBM cells [35]. On the other hand, the interrelations between cell bioenergetics and GMT-related GBM cell reactions to EGF have not yet been comprehensively studied in terms of ROS generation.

EGF was reported to augment the invasiveness of non-glial tumors [36,37]. In neural systems, GAM-related EGF fluxes within tumor microenvironment [10] constitute the positive feedback loop between inflammation, EGF secretion, and GBM progression. Considering the postulated role of the EGF in GBM pathology, we hypothesized that EGF-dependent pro-invasive signaling induces phenotypic and metabolic reprogramming of GBM cells towards the invasive phenotype. This can preserve the bioenergetic homeostasis of GBM and activate signaling pathways that further augment GBM invasion. To verify this notion, we comprehensively estimated T98G cell reactions to the EGF in vitro and correlated them with the changes of the cell metabolic profile.

## 2. Results

### 2.1. EGF Induced Proliferation, Metabolic Activity, and Morphological Changes in GBM Cells

**Figure 1:**
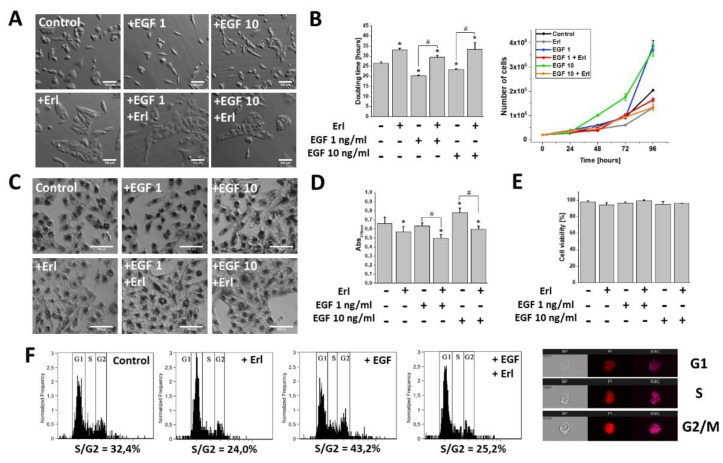
Effect of EGF (Epidermal Growth Factor) on GBM (Glioblastoma multiforme) cells’ morphology and proliferation activity. (**A**) EGF promoted development of a spindle-like cellular phenotype after only 24 h of incubation. Note the attenuation of this effect upon EGFR (Epidermal Growth Factor Receptor) inhibition with erlotinib (Erl). (**B**) Effect of EGF on proliferation kinetics (doubling time) modulation within GBM cells. (**C**,**D**) Metabolic (reducing power; MTT assay) activity of GBM cells incubated with EGF and/or Erl for 48 h. Note a pronounced MTT reduction by GBM cells during EGF incubation. The 570 nm values shown in the column plot were normalized to cell number. (**E**) EGF and Erl (72 h) showed negligible pro-apoptotic effects on GBM cells. (**F**) Pro-mitotic activity of EGF within GBM cells resulted in an increased percentage of S/G2-phase cells. Images on the right show representative objects collected during cytometric cell cycle analysis in three different channels (BF: bright-field; PI: propidium iodide; SSC: side scatter). Bars represent SD, *n* = 3. Statistical significance was calculated with non-parametric Mann–Whitney test, * *p* < 0.05 vs. control; # *p* < 0.05 vs. reference condition. Scale bars = 100 µm.

EGF has long been shown to affect the development of GBM [10]. Indeed, microscopic analyses revealed noticeable phenotypic shifts towards a spindle-like (mesenchymal) morphology in the T98G cell populations exposed to the EGF (1 and 10 ng/mL). Interestingly, EGFR inhibition by Erlotinib (Erl) entirely promoted the epithelial phenotype in both the EGF-treated and non-treated cell populations. These effects were accompanied by a pronounced dose-dependent pro-proliferative activity of the EGF (Figure 1A,B). Furthermore, microscopic and spectrophotometric experiments revealed intensified MTT substrate processing in the cells exposed to 48 h EGF treatment. This was manifested by a considerable accumulation of formazan crystals within the intracellular space (Figure 1C,D). Concomitantly, the effects of EGF on the cell morphology, proliferation, and metabolism were inhibited by erlotinib administration (Erl, a chemical inhibitor of the EGF receptor (EGFR); Figure 1A–D). Notably, no cytotoxic/pro-apoptotic effects of EGF/Erl were observed, as demonstrated by ca. 100% cell viability in all experimental variants (Figure 1E). This notion was confirmed by the DNA content analyses, which showed a strong pro-mitotic activity of the EGF, illustrated by a prominent increase of S and G2/M fractions after 24 h EGF treatment (Figure 1F). Again, the inhibition of EGFR signaling by Erl resulted in G1 arrest of GBM cells. These data show that EGF induced proliferation and an invasive morphology of GBM cells in an EGFR-dependent manner.

### 2.2. EGF Augmented T98G Cell Motility and Intracellular ATP/Lactate Production

**Figure 2:**
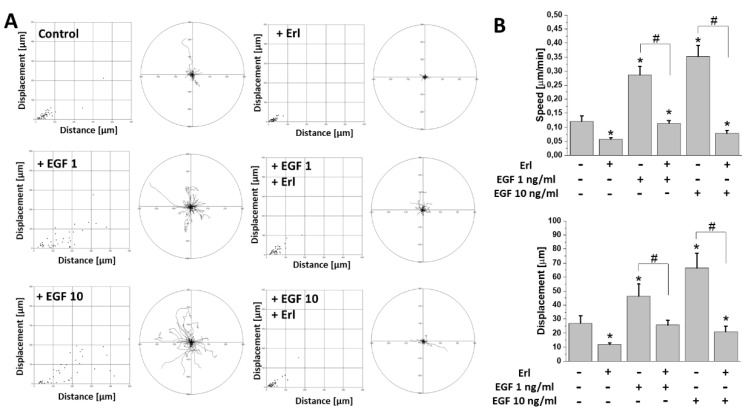
EGF augmented migration activity of GBM cells in vitro. (**A**) The effect of 72 h exposure of GBM cells to EGF and/or Erl. Dot plots represent displacement (X axis) and total length of trajectory (Y axis) calculated for single analyzed cells. Circular plots depict trajectories of individual cells. (**B**) Quantitative analysis of parameters (speed, displacement) describing efficiency of cells’ migration activity changes in examined conditions. Note the highly pronounced stimulation of cellular motility by EGF. Bars represent S.E.M.; *n* = 40. Statistical significance was calculated with non-parametric Mann–Whitney test, * *p* < 0.05 vs. control; # *p* < 0.05 vs. reference condition.

GBM cells efficiently invade the adjacent brain regions. To estimate the effect of EGF on the invasiveness of T98G cells, we performed time-lapse video microscopy analyses of their motility in the presence of EGF. Our data indicated a prominent pro-migratory activity of EGF at both applied concentrations (Figure 2A). This was illustrated by the increased (>200%) cell motility (cell speed and displacement) in the populations of EGF-stimulated cells in comparison to controls (Figure 2B). Again, Erl completely abolished this effect, decreasing the motility of T98G cells cultivated in the absence of EGF.

**Figure 3:**
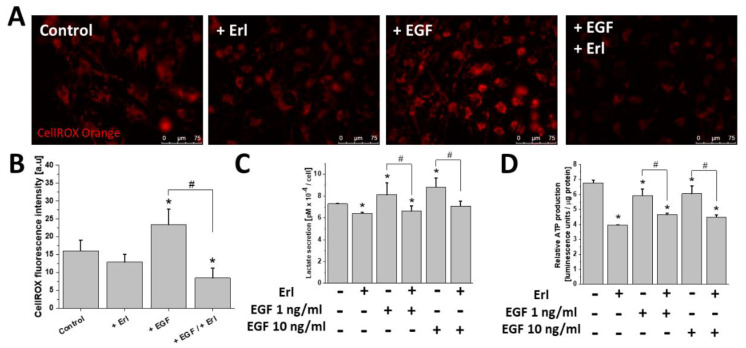
Effect of EGF on cellular redox and bioenergetic status modulation. (**A**,**B**) EGF (10 ng/mL) promoted mitochondrial (mt) ROS production. Measurements were performed with CellROX Orange reagent. (**C**,**D**) EGF noticeably affected the efficiency of lactate/ATP biosynthesis in GBM cells. Note that all of the abovementioned phenomena were attenuated by EGFR inhibition with erlotinib. Bars represent S.E.M, *n* = 40 cells. Statistical significance was calculated with non-parametric Mann–Whitney test, * *p* < 0.05 vs. control; # *p* < 0.05 vs. reference condition. Scale bars = 75 µm.

Furthermore, we examined the effect of the EGFR-dependent signaling on cellular metabolic homeostasis, and in particular on the mitochondrial ROS, ATP, and lactate production in the EGF-treated T98G cells (Figure 3). EGF-exposed cells showed a pronounced ROS upregulation (Figure 3A,B). This phenomenon was accompanied by a prominent modulation of lactate/ATP production. Specifically, the increase of lactate secretion (Figure 3C) was accompanied by a slight reduction of ATP levels within the motile EGF-treated cells (Figure 3D). This effect (as well as ROS production) was abolished by the application of Erl. EGFR inhibition also considerably perturbed the production of ATP/lactate in T98G cells, regardless of culture conditions. Thus, links exist between the EGF-dependent augmentation of invasiveness, metabolic reprogramming, and ROS production in T98G cells.

### 2.3. EGF Induced Cytoskeletal Rearrangements and Invasiveness of T98G Cells

**Figure 4:**
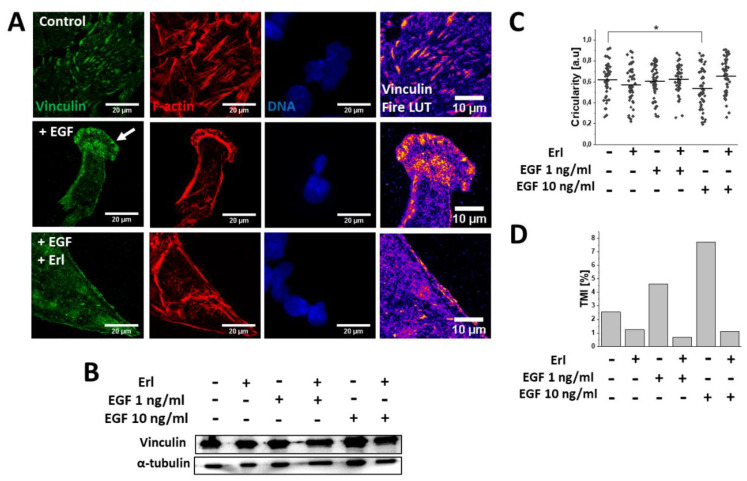
EGF (10 ng/mL; 72 h) promoted F-actin-/vinculin-related cytoskeletal rearrangements within GBM cells. (**A**) Visualization of EGF-dependent F-actin remodeling accompanied by vinculin redistribution towards the leading edges of GBM cells. Fire LUT mask depicts accumulation of vinculin within cellular compartments (blue: low level, yellow/orange: high level). (**B**) Western blot examination of total intracellular vinculin content changes in GBM cells during EGFR signaling modulation. α-tubulin was detected as a reference protein. (**C**) EGF promoted transition towards mesenchymal (spindle-like; lower circularity) morphology in a dose-dependent manner. (**D**) EGF augmented the transmigration potential of GBM cells (expressed as TMI (transmigration index)) in in vitro Transwell^®^ assay. Note that EGF exerted negligible effects on vinculin level changes but prominently enhanced invasive properties of GBM cells. *n* = 40 cells. Statistical significance was calculated with non-parametric Mann–Whitney test, * *p* < 0.05 vs. control.

To further examine the mechanisms underlying the pro-invasive effects of the EGF, we performed detailed microscopic analyses of the actin cytoskeleton architecture in the EGF-treated cells. TIRF (Total Internal Reflection Fluorescence) microscopy revealed a substantial remodeling of F-actin filament architecture and focal adhesions within the T98G cells in response to the EGF (10 ng/mL) treatment. In particular, spindle-like (mesenchymal) cells with less prominent stress fibers, but pronounced F-actin polymerization regions in the leading edges were observed (Figure 4A). Moreover, dispersed aggregates of vinculin instead of vinculin + plaque-like structures were present in the leading edges of the EGF-stimulated GBM cells (Figure 4A). This phenomenon was correlated with the significant decrease of cellular circularity at the population level after 72 h EGF (10 ng/mL) exposure (Figure 4C), and with the prominent increase of the T98G transmigration potential (TMI; Figure 4D). Interestingly, leading-edge formation (Figure 4A) and the induction of T98G invasiveness (Figure 4D) were abolished by Erl in the EGF-treated T98G cells. Erl treatment also led to the disorganization of the F-actin cytoskeleton and focal adhesions’ sides, accompanied by their morphological switch into an epithelioid phenotype. In turn, the Western blot analysis did not reveal any changes in the intracellular vinculin levels under EGF/Erl stress (Figure 4B). Collectively, these observations confirmed the involvement of EGFR-dependent signaling in determination of T98G cell invasiveness.

### 2.4. ROS Upregulation Was Responsible for EGF-Induced T98G Motility 

**Figure 5:**
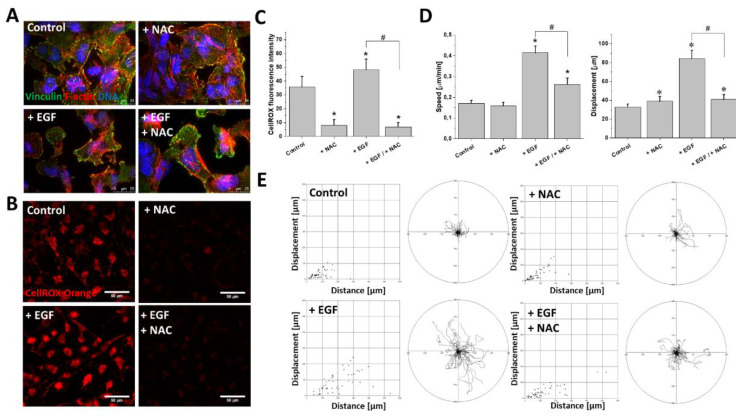
Effect of ROS scavenger N-acetyl-L-cysteine on EGF-induced enhancement of migration activity of GBM cells. (**A**) Decreased ROS levels in GBM cells did not attenuate EGF-evoked changes towards spindle-like morphology. (**B**,**C**) NAC (1 mM) strongly decreased mtROS production in examined GBM cells. (**D**,**E**) Effect of NAC on GBM cells’ motility in the presence/absence of EGF (10 ng/mL). Note the considerable attenuation of pro-migratory EGF activity upon intracellular ROS depletion. Bars represent S.E.M, *n* = 40 cells. Statistical significance was calculated with non-parametric Mann–Whitney test, * *p* < 0.05 vs. control; # *p* < 0.05 vs. reference condition. Scale bars = 25 µm (**A**) and 50 µm (**B**).

Finally, we focused on the significance of ROS upregulation for EGF-induced T98G invasiveness. Interestingly, the administration of N-Acetyl-L-cysteine (NAC; 1 mM) did not abolish the EGF-induced morphological changes of the T98G cells and did not inhibit the pro-invasive remodeling of the F-actin cytoskeleton (Figure 5A). On the other hand, NAC remarkably attenuated the EGF-induced upregulation of ROS (Figure 5B,C). This effect was accompanied by a significant inhibition of the EGF-induced T98G motility. At the population level, this was illustrated by a decrease of the average speed of motility and displacement of the EGF/NAC-treated cells (Figure 5D). Accordingly, single cell trajectories of EGF/NAC-treated cells clearly demonstrated a retardation of their motility in comparison to the EGF-stimulated cells (Figure 5E). Notably, NAC had a negligible impact on T98G motility when administered alone (Figure 5D,E). These data show the cooperation of the ROS-dependent and ROS-independent EGFR-dependent pathways that regulate T98G motility and cytoskeleton architecture, respectively.

## 3. Discussion

The growth of glioblastoma multiforme (GBM) depends on complex neurochemical signaling networks within the brain tissue. During GBM progression, numerous growth factors are secreted by microglia and/or neural cells that reside in the tumor microenvironment. They result in GBM growth enhancement and in the stimulation of its malignancy. In particular, EGF is involved in the response of brain tissue to pathological processes [20,22,38]. Considering this phenomenon, it is worth emphasizing that the potential of GBM to penetrate and deform healthy brain tissues during invasion [39,40,41] can be related to EGF secretion in the absence of constitutively activated EGF receptors in GBM cells (EGFR type III or EGFRvIII [13]). This may occur during surgical intervention within the tumor area. Moreover, many reports have raised the matter of EGF receptor (EGFR) overexpression in GBM cells and its correlation with GBM malignancy [42]. Taking into account these issues, we focused on the role of the EGF in the regulation of GBM progression and invasion. In our study, EGF induced metabolic reprogramming and enhanced the invasive potential of GBM cells in a manner partly dependent on the concomitant ROS production. Malignant GBM cells must negotiate numerous mechanical obstacles to invade neighboring brain tissue. An effective GBM invasion requires a permissive (elastic) cytoskeleton architecture that determines the directional motility of the cells and their deformability, facilitating their penetration of narrow tissue obstacles and the colonization of distant brain structures [26]. Examination of cytoskeletal rearrangements within the EGF-treated cells revealed a distinct remodeling of the actin cytoskeleton and the redistribution of vinculin towards the cellular leading edges. These effects were accompanied by increased T98G cell motility and enhancement of the GBM cell transmigration potential (TMI). The suppression of pro-invasive (i.e., EMT-related) signaling within GBM cells after chemical EGFR inhibition (by Erl) confirmed the involvement of EGFR signaling in GBM progression [28]. Notably, acquisition of the epithelial phenotype by the Erl-treated GBM cells may also suggest the involvement of other EGFR ligands in determination of the GBM invasive phenotype. Finally, studies on the bioenergetic profiles of GBM cells exposed to EGF revealed considerable changes in the ATP/lactate production. This observation strongly correlates with their enhanced migratory and proliferative activity, which requires considerable amounts of energy in the form of ATP/GTP. Reduction of the ATP and lactate level after erlotinib treatment confirmed the role of the EGFR in the induction of aerobic glycolysis (the Warburg effect). It may also explain the inhibition of the GBM invasiveness as an effect of energy depletion in the absence of the EGF. On the other hand, lower intracellular ATP levels in the EGF-treated cells, accompanied by enhancement of lactate secretion, indicated the increased energy consumption in these cells, compensated by the EGF-induced glycolysis.

In our study, NAC application did not prevent the EGF-induced T98G morphology/cytoskeleton rearrangements. However, it remarkably abolished the EGF-induced triggering of T98G cell motility. This observation indicated the inability of cells to effectively respond to EGF signals in reducing conditions. Concomitantly, it underlined the role of ROS in the regulation of GBM cell invasiveness, justifying a combined application of antioxidants and EGF inhibitors in the GBM therapy [41]. Cellular redox status and the cytosolic distribution of ROS are key players in the regulation of the cytoskeleton dynamics and cellular motility [32]. Notably, their functions depend on the activity of numerous molecular executors, including cytoskeleton-bound small G proteins (incl. Rac1). Furthermore, mitochondria have been reported to depend on the EGFR, and this sensitivity is crucial for metabolic homeostasis of cancer cells. EGFR activation is also related to NOX (NADPH oxidase) [16] mobilization and superoxide production. Our current observations indicated the role of the EGF-induced shift of mitochondrial function towards ROS generation, which is compensated by the induction of energy production by aerobic glycolysis.

Collectively, we present data that confirm the essential role of the EGF in the stimulation of GBM cell invasion and shed light on the role of ROS in this effect. Apparently, EGF induces metabolic reprogramming and ROS production in GBM cells in an EGFR-dependent manner. Collaborative ROS/EGFR-dependent signaling further augments GBM cells’ invasiveness in hypoxic conditions. In the context of the reports of EGFR upregulation in GBM cells, our data point to combined EGFR/ROS targeting as a way to interfere with GBM progression. On the other hand, our data also indicate the need for the new treatment strategies that could combine an anti-EGFR/ROS approach with the application of metabolic inhibitors. The additional metabolic approach might prevent the microevolution of drug resistance and reduce the side-effects of conventional therapies. However, all extrapolations to clinical conditions and correlations with disease progression require further research on in vivo models.

## 4. Materials and Methods 

### 4.1. Cell Culture

Human glioblastoma multiforme T98G cells (ATCC, CRL-1690) were cultured in standard conditions with high levels of glucose (4500 g/L) DMEM medium (Sigma), supplemented with 10% fetal bovine serum (FBS; Gibco) and 1% antibiotic-antimycotic Solution (Sigma) as described previously [43]. For each experiment, cells were harvested with Ca^2+/^Mg^2+^-free PBS (Corning) with 0.5 mM EDTA (UltraPureTM; Invitrogen) solution, counted in a Z2 particle counter (Beckman Coulter), and seeded at an appropriate density into multi-well tissue culture plates (Eppendorf). Human epidermal growth factor (EGF; Sigma; No. E9644) was dissolved in 10 mM acetic acid/0.1% BSA and stored at −80 °C. EGFR inhibitor erlotinib (Erl; Sigma; No. SML2156) stock solution (4.6 mM; 9.2 µM in cell culture media) was prepared in DMSO (Sigma). N-Acetyl-L-cysteine (NAC; Sigma; No. A9165) was dissolved in sterile-filtered H_2_O to 1 M and used in cell culture at 1 mM final concentration.

### 4.2. Cell Proliferation, Viability, and MTT Assays

Cells were seeded onto 12 or 24 well plates (Eppendorf) at the density of 2 × 10^4^ cells/cm^2^, cultivated until full adherence and treated with 1–10 ng/mL EGF, 9.2 µM erlotinib, or their combination. After 24–96 h, the cells were harvested and counted in a Z2 particle counter. The obtained data were processed in Origin 2020 software to calculate the kinetics of proliferation (growth rate and doubling time). The viability of the cells was estimated with a Trypan blue (Sigma; No. T8154) assay using a Burker hemocytometer, as described previously [44]. An MTT assay was applied to estimate the changes in relative cellular metabolic activity. Cells were seeded onto 96 well plates (Eppendorf) at a density of 5 × 10^3^, treated with EGF and/or erlotinib for 48 hours, followed by the addition of thiazolyl blue tetrazolium bromide (Sigma; No. M5655) water solution to each well (0.5 mg/ml) and incubation for 2–3 h at 37 °C. Afterwards, cells were observed in bright-field using Leica DMI6000B microscope in order to visualize intracellular formazan accumulation, followed by cell dissolution with isopropanol and 570 nm absorbance measurements (MultiskanTM FC Microplate Reader; Thermo Fisher Scientific).

### 4.3. Cell Migration and Transmigration 

Cells were seeded and treated as described above, and time-lapse imaging was performed 24 h and 72 h after the administration of EGF/Erl/NAC [44]. Briefly, the movement of cells was recorded for 8 h at 5 min time intervals using a Leica DMI6000B microscope equipped with an integrated modulation contrast (IMC), CO_2_ chamber (5% CO_2_), and temperature (37 °C) monitoring system. Obtained images were analyzed in Hiro v.1.0.0.4 (written by W. Czapla) by manual cell trajectory tracking, followed by the calculation of cell motility parameters (speed (µm/min) and displacement (µm)). The obtained data were additionally used for visual analysis of morphological changes towards a mesenchymal-like shape (invasive phenotype). Invasive potential of cells was examined via transmigration assay (Transwell^TM^ 8 µm micropore diameter inserts; Corning). Cells were seeded onto the top of a micropore membrane at a density of 2 × 10^4^ cells/cm^2^, and allowed to transmigrate for 24–72 h in the presence of EGF and/or erlotinib and to proliferate for next 72 h. The specimens were then subjected to time-lapse videomicroscopy (8 h, 5 min intervals; as above), followed by cell harvesting and counting in a Z2 particle counter. Transmigration index (TMI) was expressed as a percentage of cells that managed to penetrate the micropore membrane over the course of the experiment time (24–72 h) in relation to the proliferation rates in each condition [41].

### 4.4. Immunocytochemistry

The cells were cultured in 12 well plates on UVC-sterilized coverslips and treated with the relevant concentrations of EGF/Erl/NAC for 72 h. For immunolocalization, the cells were fixed with 3.7% formaldehyde, followed by 0.1% Triton X-100 permeabilization. Blocking of non-specific binding sites was performed with 2% BSA incubation for 30–45 min in 37 °C. Specimens were incubated for 45 min with monoclonal IgG mouse anti-vinculin (Sigma; No. V9131; 1:300) antibody diluted in 2% BSA and 0.01% Tween. After washing (2% BSA), the sets of secondary antibodies/specific dyes were applied for 45 min: AlexaFluor488-conjugated donkey anti-mouse IgG (Invitrogen; No. A21202), AlexaFluor546-conjugated phalloidin (Invitrogen, No. A22283) for F-actin visualization, and Hoechst 33258 (Sigma) for DNA staining. Images were acquired with a Leica DMI6000B fluorescence microscope equipped with a DFC360FX CCD camera and total internal reflection fluorescence (TIRF) module. Z-stack scanning followed by 2D deconvolution (LAS X; Leica) were performed for high-resolution vinculin/cortical F-actin co-localization. Raw images were additionally processed (contrast adjustment, background subtraction, fluorimetric analysis, circularity calculation) in ImageJ software.

### 4.5. Immunoblotting 

For the estimation of GBM invasion/cytoskeleton-related vinculin rearrangements, the cells subjected to 72 h incubation with EGF/erlotinib were harvested with cold (~4 °C) Ca^2+^/Mg^2+^-free PBS/EDTA solution, centrifuged, and dissolved in cell lysis buffer with protease inhibitor cocktail, followed by the freeze–thaw procedure and sonication. A Bradford assay was used for determination of the total protein content in the obtained lysates. Samples (20 µg of protein) were separated on 12% polyacrylamide gel (SDS-PAGE electrophoresis; Laemmli protocol [31]), followed by electrotransfer onto PVDF membranes (Immun-Blot^®^ PVDF Membrane, #1620177; Bio-Rad, Hercules, CA). Membranes were blocked with skimmed milk/TBST solution. For immunodetection, the monoclonal IgG mouse anti-vinculin (Sigma; No. V9131; 1:500) was used. α-tubulin, labeled with monoclonal IgG mouse anti-α-tubulin antibody (Sigma; No. T9026; 1:1000) was used as a reference protein. Signal detection was performed with HRP-conjugated antibodies: HRP-conjugated goat anti-mouse IgG (Thermo Fisher Scientific; No. 31430), followed by chemiluminescent HRP substrate incubation (Merck, Luminata Crescendo; No. WBLUR0500). For membrane imaging, a MicroChemi system (SNR Bio-Imaging System) was used.

### 4.6. Mitochondrial ROS Production Detection

Cells were seeded onto dedicated 24 well fluorescence-imaging plates (Eppendorf, No. 0030741005) and treated with EGF/Erl/NAC for 24 h. For the determination of mitochondrial ROS levels, the cells were incubated in the presence of 1 µl/ml of CellROX Orange reagent (Invitrogen; No. C10443) for 30 min, immersed in FluoroBriteTM DMEM medium (Gibco; supplemented with 10% FBS and 1% GlutaMax [43]) and examined with a Leica DMI6000B microscope (ex. 546 nm) equipped with a CO_2_ (5%) chamber at 37 °C. Fluorimetric analyses (fluorescence intensity, background subtraction) of the obtained images were performed in ImageJ software.

### 4.7. Image Cytometry and Cell Cycle Analysis

Cells were seeded onto 6 well plates at a density of 10^4^/cm^2^ and treated with EGF and/or Erl for 48 h. For cell cycle analyses, FxCycle™ PI/RNase Staining Solution (Invitrogen; No. F10797) was applied. Cells were harvested with 0.25% Trypsin/EDTA solution, centrifuged, and fixed with 3.7% formaldehyde for 10 min at room temperature. After double washing with PBS and centrifugation, the cells were resuspended in staining solution for 30 minutes and incubated overnight in darkness. Signal detection was performed with ImageStreamX cytometer (Merck) [41,43]. For each condition, at least 1000 singlets were collected. The data were processed using IDEAS 6.2 software (Merck).

### 4.8. ATP and Lactate Production Measurements

The cells were seeded onto 12 well plates at a density of 5 × 10^4^ cells/cm^2^ (ATP measurement) or 2 × 10^4^ cells/cm^2^ (lactate measurement) and treated with EGF/Erl for 2 h or 24 h. For the estimation of intracellular ATP production, an ATP determination kit was applied (Invitrogen; No. A22066). The cells were lysed with cold distilled water (300 µL/well). Collected lysates (1.5 ml tubes) were heated at 95 °C for 10 minutes, followed by cooling on ice (1–2 min) and centrifugation. Subsequently, the samples were mixed at the ratio of 1:9 (final volume = 100 µL) with the standard reaction solution in 96 well black plates with glass bottoms (Eppendorf). Bioluminescence detection was performed with the Infinite 200 Pro reader (Tecan). Extracellular (medium) lactate levels were examined using a Lactate Assay Kit (Sigma; No. MAK064). Medium samples were diluted in PBS at the ratio of 1:100, followed by deproteinisation (LDH removal) with 10 kDa NMWCO Amicon^®^ Ultra centrifugal filters (Sigma; No. UFC5010). The whole procedure was performed on ice. After the addition of reaction solution at the ratio of 1:1 (final volume = 100 µL) on a 96 well microplate (Sarstedt), lactate levels were evaluated at 570 nm with MultiskanTM FC Microplate Reader (Thermo Fisher Scientific).

### 4.9. Statistical Analysis

Statistical significance of differences was tested with non-parametric Mann–Whitney U-test. Analyses were performed using Origin 2020 software.

## 5. Conclusions

Our data confirmed the EGFR-dependent pro-neoplastic and pro-invasive activity of EGF in human GBM. Interrelations of EGF-dependent pro-invasive signaling with energy metabolism seem to be crucial for GBM progression, because they apparently preserve the bioenergetic homeostasis of GBM cells in hypoxic regions of brain tissue. These interrelations depend on metabolic reprogramming of GBM cells and are executed by the interspersed ROS-dependent/-independent pathways. Thus, we have provided insight into the vicious cycle between GBM invasion and the disruption of brain tissue, which depends on the abnormal secretion of the epidermal growth factor (EGF) by non-neuronal cells (e.g., glioma-associated microglia). This paracrine loop is an attractive target for anti-GBM therapies.

## Abbreviations

EGF	Epidermal Growth Factor
EGFR	Epidermal Growth Factor Receptor
Erl	Erlotinib
GBM	Glioblastoma multiforme
HRP	Horseradish Peroxidase
NAC	N-acetyl-L-cysteineReactive Oxygen Species
ROS	Reactive Oxygen Species
mtROS	Mitochondrial Reactive Oxygen Species
TIRF	Total Internal Reflection Fluorescence
